# Risk of supranormal left ventricular ejection fraction in patients with aortic stenosis

**DOI:** 10.1002/clc.24255

**Published:** 2024-03-12

**Authors:** Naoya Inoue, Hayato Ohtani, Shuji Morikawa, Yohei Takayama, Takashi Ogane, Takehiro Hiramatsu, Hiroki Kumihashi, Toyoaki Murohara

**Affiliations:** ^1^ Department of Cardiology Chutoen General Medical Center, Kakegawa Shizuoka Japan; ^2^ Department of Cardiology Nagoya University Graduate School of Medicine Nagoya Aichi Japan; ^3^ Division of Cardiology, Internal Medicine III Hamamatsu University School of Medicine Shizuoka Hamamatsu Japan

**Keywords:** all‐cause mortality, aortic stenosis, aortic valve area, heart failure, supranormal left ventricular ejection fraction

## Abstract

**Background:**

Cardiovascular events are increasing in patients with supranormal left ventricular ejection fraction (snLVEF). However, the effect of snLVEF in patients with aortic stenosis (AS) remains unclear, especially in patients with moderate AS.

**Hypothesis:**

This study aimed to evaluate the prognosis of mortality and heart failure (HF) in patients with LVEF ≥ 50% and moderate or severe AS.

**Methods:**

This retrospective study targeted patients with moderate or severe AS and LVEF > 50%. LVEF of 50%–65% was classified as normal LVEF (nLVEF, nEF group) and >65% as snLVEF (snEF group). AS severity was stratified based on the aortic valve area into moderate (1.0–1.5 cm²) and severe (<1.0 cm²). Primary outcomes included all‐cause mortality and HF hospitalization.

**Results:**

A total of 226 participants were included in this study. There were 67 and 65 participants with moderate AS in snEF (m‐snEF) and nEF groups (m‐nEF), respectively, and 41 and 53 participants with severe AS in the snEF (s‐snEF) and nEF groups (s‐nEF), respectively. During the observation period (median: 554 days), the primary composite outcome occurred in 108 individuals. Cox hazard analysis revealed no significant differences among the four groups in primary composite outcomes. With respect to HF hospitalization, the adjusted hazard ratios (95% confidence intervals) with m‐snEF as the reference were as follows: m‐nEF, 0.41 (0.19–0.89); s‐nEF, 1.43 (0.76–2.67); and s‐snEF, 1.83 (1.00–3.35).

**Conclusions:**

The risk of HF hospitalization for m‐snLVEF was higher than m‐nLVEF and not significantly different from s‐nLVEF.

## Graphical abstract

In patients with moderate or severe aortic stenosis (AS), supranormal left ventricular ejection fraction (LVEF; LVEF > 65%) may increase the risk of heart failure hospitalization. The risk did not differ significantly between moderate AS + supranormal LVEF and severe AS + normal LVEF cases.

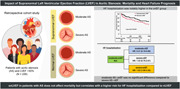

## 1 INTRODUCTION

The prevalence of cardiovascular disease in Japan and other countries is increasing with the increase in the aging population.^1^, ^2^ Among cardiovascular diseases, valvular diseases are the fifth highest cause of death,^3^ with a concerning risk of sudden death associated with severe aortic stenosis (AS).^4^ The prevalence of severe AS is 12.4% across all age groups and 3.4% in those aged ≥75 years,^5^ making it a common condition. In addition to surgical aortic valve replacement (AVR), in recent years, transcatheter AVR has been introduced, allowing AVR to be performed in older patients, leading to improvements in mortality rates.^6^, ^7^

The timing of AVR has traditionally been determined based on the presence of symptoms associated with AS and its severity as assessed by echocardiography.^8^ However, it is recommended to comprehensively assess these indications rather than relying on a single criterion.^8^ Khan et al.^9^ reported that heart failure (HF) in cases of moderate AS complicated by reduced left ventricular ejection fraction (LVEF) is associated with higher mortality and HF hospitalization rates compared to HF without AS. In some cases, conditions other than severe AS may also be associated with poor prognosis. Moreover, Franke et al. suggested that performing AVR in cases of moderate AS improves prognosis.^10^

It has been suggested that LVEF decreases under the influence of the afterload during the progression of AS,^8^ and an LVEF ≤ 50% is often used in the treatment guidelines for patients with AS.^8^ Recently, a new category called supranormal LVEF (snLVEF; ejection fraction [EF] >65%–70%) has been proposed in the field of HF,^11^ and several reports have indicated a poor prognosis, including mortality, in patients with snLVEF suffering from HF.^12^, ^13^ However, to date, studies examining the relationship between LVEF and the severity of AS are limited. In a study by Imamura et al.,^14^ patients with severe AS and snLVEF showed poorer outcomes after AVR than those with severe AS and normal LVEF (nLVEF; EF 50%–64%). However, this study only focused on cases of severe AS, which already warrants consideration for AVR. Hence, the impact of this study on the treatment guidelines may not be significant.

The prognostic influence of snLVEF in moderate AS, for which AVR is currently not indicated, has not been evaluated previously. Investigating the prognosis of such cases may have significant implications for the reevaluation of indications for AVR in the future and thus holds valuable research potential. Therefore, this study aimed to evaluate the prognosis of mortality and HF in patients with LVEF ≥ 50% and with moderate or severe AS.

## 2 METHODS

This retrospective observational study was performed in accordance with the standards of the Declaration of Helsinki and current ethical guidelines. This study was approved by the Ethics Committee on Medical Research of the Chutoen General Medical Center (Reference Number: 1260231201). Informed consent was obtained using an opt‐out approach, and information about this study (opt‐out statement) was published on the hospital's website according to the Personal Information Protection Law. In cases where requests were made by patients or their families, the relevant information was removed from the research subject.

### 2.1 Study design, participants, and measurements

This study included patients who underwent echocardiography at Chutoen General Medical Center between May 2013 and November 2020. Patients diagnosed with AS who were ≥18 years of age and had LVEF ≥ 50% were identified. Data on the aortic valve area (AVA), maximum blood flow velocity across the aortic valve (Vmax), and the mean pressure gradient (mPG) across the aortic valve were extracted when available. Only echocardiography data from the earliest date were used in cases with duplicate data from the same patient. The exclusion criteria were as follows: (1) missing measurements for AVA, Vmax, or mPG; (2) suggestion of mild AS with AVA > 1.5 cm²; (3) history of AVR; (4) presence of obstructive hypertrophic cardiomyopathy; and (5) lack of follow‐up at the hospital after echocardiography.

Patients who met the inclusion criteria were categorized as follows: patients with LVEF of 50%–65% were classified as having nLVEF (nEF group), and those with LVEF > 65% as having snLVEF (snEF group).^14^ AS severity was stratified based on AVA as follows: moderate AS (AVA,1.0–1.5 cm²) and severe AS (AVA < 1.0 cm²).^9^ The use of AVA for stratification was justified by the fact that, even with preserved LVEF, a small left ventricular volume can lead to a decrease in stroke volume (SV), resulting in a low‐pressure gradient.

The study population was further classified into the following four groups: m‐snEF (moderate AS and snEF), s‐snEF (severe AS and snEF), m‐nEF (moderate AS and nEF), and s‐nEF (severe AS and nEF).

### 2.2 Primary composite outcome

Patients identified from the echocardiographic data were retrospectively followed up until November 2023 (maximum follow‐up duration: 1500 days), with all‐cause mortality and HF hospitalization defined as the primary composite outcomes. The criteria for hospitalization due to HF were based on previous reports and included worsening of typical HF symptoms and radiographic congestion, elevated natriuretic peptides and filling pressures on echocardiography, and initiation of intravenous diuretic therapy.^15^, ^16^ If HF hospitalization occurred at the time of echocardiography, subsequent hospitalization due to HF was considered an endpoint event. In the case of repeated hospitalization for HF, only the first hospitalization was recorded. HF hospitalization as a primary outcome was censored at the time of surgical AVR or transcatheter AVR; however, patients were monitored for survival after AVR.

### 2.3 Statistical analysis

Categorical variables were analyzed using the chi‐square or Fisher's exact test and are presented as numbers and percentages. For continuous variables, after evaluating normality, parametric or nonparametric tests (Kruskal–Wallis tests) were performed. Time‐to‐event rates for mortality and HF after echocardiography were calculated and group‐wise comparisons of the event occurrence curves were conducted using the log‐rank test. When a significant difference was observed in the log‐rank test among the four groups, multiple comparisons were performed using the Bonferroni method. Univariate Cox proportional hazards analysis was performed for the primary composite and individual outcomes, followed by multivariate analysis using a forced entry method for factors potentially influencing clinical outcomes. Statistical significance was set at *p* < .05. All statistical analyses were conducted using R Statistical Software (Foundation for Statistical Computing) or EZR (Jichi Medical University).^17^ To identify associations between HF hospitalization and LVEF, we employed restricted cubic splines. We conducted a complete case analysis by examining only samples with no missing values in the data necessary for each statistical analysis during the patient characteristic comparisons. For Cox hazard analysis, there were 11 missing data points for body mass index (BMI) and 18 for B‐type natriuretic peptide (BNP); consequently, we performed analysis using the data imputed through multiple imputations using the mice function in the R package to account for missing values.

## 3 RESULTS

### 3.1 Comparison of patient characteristics

Among patients who underwent echocardiography at our hospital between May 2013 and November 2020, 226 were included in this study. Of these, 67, 41, 65, and 53 patients were included in the m‐snEF, s‐snEF, m‐nEF, and s‐nEF groups, respectively. Significant differences among the four groups were observed in sex, BMI, BNP, and echocardiographic parameters such as LVEF, AVA, mPG, and Vmax. Additionally, differences were observed in the LVDd, LVDs, and left ventricular posterior wall thickness (Table 1).

**Table 1:** Comparison of patient characteristics for analysis.

	Supranormal LVEF (snEF group, *n* = 108)			Normal LVEF (nEF group, *n* = 118)		*p*‐Value
	Moderate AS (m‐snEF group, *n* = 67)		Severe AS (s‐snEF group, *n* = 41)	Moderate AS (m‐nEF group, *n* = 65)	Severe AS (s‐nEF group, *n* = 53)	
Age, y	83.0 ± 7.9		85.7 ± 7.2	83.8 ± 8.2	84.6 ± 8.5	.22
Sex, Men	27 (40.2)		7 (17.0)	28 (43.0)	18 (33.9)	.031
BMI, kg/m^2^	19.4 [16.8–23.4]		18.4 [16.0–20.1]	19.7 [18.6–22.1]	19.7 [17.9–22.7]	.047
Smoking status						
Current	8 (11.9)		4 (9.7)	7 (10.7)	6 (11.3)	>.99
Former	14 (20.9)		2 (4.9)	12 (18.5)	5 (9.4)	.062
Laboratory data						
BUN, mg/dL	20.8 [16.4–28.2]		19.4 [13.6–30.5]	20.8 [15.7–30.1]	20.8 [16.2–30.7]	.80
Creatinine, mg/dL	0.86 [0.69–1.30]		0.94 [0.70–1.09]	0.89 [0.72–1.52]	0.96 [0.73–1.30]	.83
eGFR, mL/min/1.73 m^2^	51.1 [31.7–68.2]		46.4 [35.9–60.7]	50.1 [32.7–60.5]	46.3 [30.0–63.0]	.85
HbA1c, %	5.8 [5.5–6.4]		5.8 [5.5–6.0]	5.8 [5.6–6.2]	5.6 [5.2–6.5]	.42
BNP, pg/mL	193 [91–384]		313 [114–571]	213 [73–753]	439 [160–1215]	.004
LDL‐C, mg/dL	102 [80–122]		89 [74–104]	96 [79–109]	102 [82–132]	.12
Medical history						
Hypertension	45 (67.1)		25 (60.9)	39 (60.0)	33 (62.2)	.84
Diabetes mellitus	16 (23.8)		9 (21.9)	14 (21.5)	13 (24.5)	.98
Dyslipidemia	14 (20.8)		10 (24.3)	12 (18.4)	12 (22.6)	.88
Atrial fibrillation	13 (19.4)		16 (39.0)	15 (23.0)	14 (26.4)	.15
Prior CAD	8 (11.9)		4 (9.7)	10 (15.3)	9 (16.9)	.73
Prior heart failure	26 (38.8)		20 (58.7)	32 (49.2)	28 (52.8)	.44
Prior stroke	5 (7.4)		6 (14.6)	6 (9.2)	5 (9.4)	.67
Hemodialysis	8 (11.9)		2 (4.8)	6 (9.2)	4 (7.5)	.67
Treatment at baseline						
Beta‐blockers	14 (20.8)		9 (21.9)	19 (29.2)	11 (20.7)	.66
CCB	40 (59.7)		17 (41.4)	31 (47.6)	21 (39.6)	.12
ACE inhibitors	4 (5.9)		4 (9.7)	3 (4.6)	4 (7.5)	.77
ARB	32 (47.7)		12 (29.2)	20 (30.7)	19 (35.8)	.14
Diuretics	25 (37.3)		18 (43.9)	27 (41.5)	23 (43.3)	.87
Statins	15 (22.3)		11 (26.8)	15 (23.0)	10 (18.8)	.83
Echocardiographic data						
LVEF, %	71 [68–73]		69 [67–73]	60 [57–62]	60 [57–63]	<.001
AVA, cm^2^	1.1 [1.0–1.3]		0.8 [0.7–0.9]	1.2 [1.1–1.3]	0.8 [0.7–0.9]	<.001
AV meanPG, mmHg	27.1 [23.7–36.9]		38.8 [31.8–48.9]	26.3 [22.0–34.8]	39.0 [27.8–53.5]	<.001
Vmax, m/s	3.4 [3.2–3.9]		3.9 [3.7–4.5]	3.3 [3.1–3.8]	4.0 [3.4–4.7]	<.001
LVDd, mm	43 [39–46]		41 [37–45]	45 [41–51]	45 [37–47]	.008
LVDs, mm	26 [24–28]		25 [23–27]	32 [28–35]	30 [27–33]	<.001
IVST, mm	10.5 [9.2–11.7]		11.2 [10.1–12.5]	10.7 [10.0–11.9]	10.8 [9.7–12.3]	.12
LVPWT, mm	10.4 [9.0–11.5]		11.5 [10.0–12.3]	10.8 [10.1–11.3]	10.7 [9.4–12.0]	.037
E/e'	15.2 [12.5–18.9]		19.7 [16.5–25.2]	16.2 [12.6–20.7]	16.5 [13.4–20.2]	.069
TR‐PG, mmHg	28.0 [24.3–37.2]		32.6 [27.5–41.7]	31.1 [24.3–36.1]	26.5 [22.2–35.4]	.087
AR > mild	29 (43.2)		21 (51.2)	41 (63.0)	29 (54.7)	.15
MR > mild	29 (43.2)		24 (58.5)	35 (53.8)	33 (62.2)	.18
MS > mild	7 (10.4)		2 (4.8)	4 (6.1)	2 (3.7)	.57

*Note*: Data are presented as mean ± standard deviation or median [interquartile range] for continuous variables and number of patients (n) and percentage (%).

Abbreviations: ACE, angiotensin‐converting enzyme; AR, aortic regurgitation; ARB, angiotensin receptor blocker; AVA, aortic valve area; BMI, body mass index; BNP, brain natriuretic protein; BUN, blood urea nitrogen; CAD, coronary artery disease; CCB, Calcium channel blocker; eGFR, estimated glomerular filtration rate; IVST, interventricular septum thickness; LDL‐C, low‐density lipoprotein cholesterol; LVEF, left ventricular ejection fraction; LVDd, left ventricular internal dimension in diastole; LVDs, left ventricular internal dimension in systole; LVPWT, left ventricular posterior wall thickness; m‐nEF, moderate aortic stenosis‐normal ejection fraction; m‐snEF, moderate aortic stenosis‐supra‐normal ejection fraction; MR, mitral regurgitation; MS, mitral stenosis; s‐nEF, severe aortic stenosis‐normal ejection fraction; s‐snEF; severe‐supra‐normal ejection fraction; TR‐PG, tricuspid regurgitation pressure gradient.

### 3.2 Impact of supranormal and normal LVEF on primary outcomes

**Figure 1:**
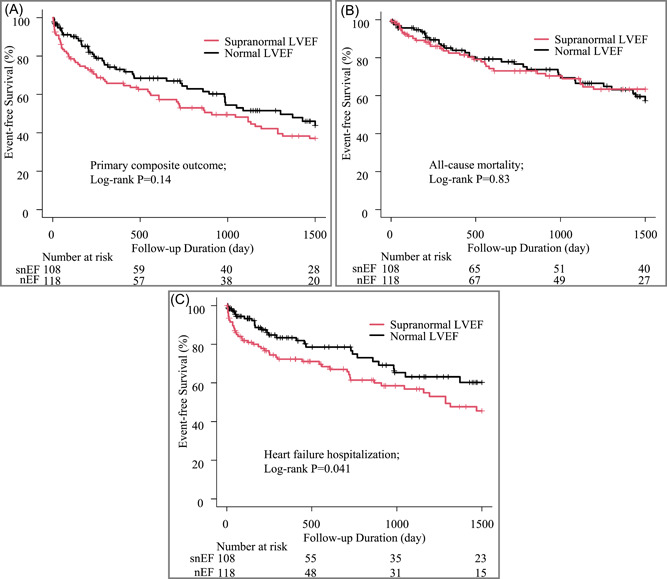
Comparison of primary outcomes between normal and supranormal left ventricular ejection fraction (A) Kaplan–Meier curves for primary composite outcomes in the normal and supranormal LVEF groups. (B) All‐cause mortality. (C) HF hospitalization. HF, heart failure; LVEF, left ventricular ejection fraction.

Log‐rank tests were conducted to compare primary outcomes between the snEF and nEF groups. During the observation period (median, 554 days), the primary composite outcome occurred in 108 patients (snEF group, 56.1% vs. nEF group, 39.8%; log‐rank *p* = .14; Figure 1A). There was no significant difference in all‐cause mortality (*p* = .83; Figure 1B). However, a significant difference was observed in HF hospitalization (*p* = .041; Figure 1C).

During the observation period, the follow‐up for HF was censored due to the implementation of AVR in 32 individuals, with 15 in the snEF group and 17 in the nEF group (*p* > .99).

### 3.3 Comparison among the four groups considering the grade of AS in relation to LVEF differences

To assess the impact of AS severity, we evaluated the primary outcomes in the four groups. The primary composite outcome rates per 100 person‐years were 24.6, 36.8, 18.4, and 28.1 in the m‐snEF, s‐snEF, m‐nEF, and s‐nEF groups, respectively (Supporting Information S1: Table S1). Detailed results were shown in Supporting Information S1: Table S1.

**Figure 2:**
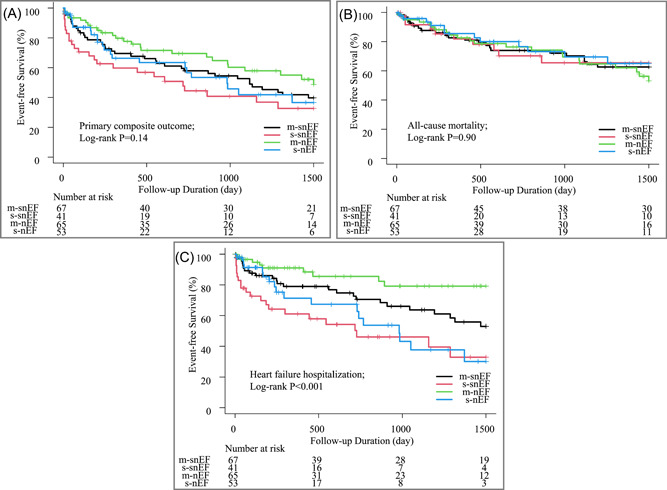
Kaplan–Meier curves for primary outcomes stratified by left ventricular ejection fraction and aortic stenosis severity. (A) Kaplan–Meier curves for primary composite outcomes in four groups. (B) All‐cause mortality. (C) HF hospitalization. HF, heart failure; LVEF, left ventricular ejection fraction; m‐nEF, moderate aortic stenosis‐normal ejection faction; m‐snEF, moderate aortic stenosis‐supranormal ejection fraction; s‐nEF, severe aortic stenosis‐normal ejection fraction; s‐snEF, severe aortic stenosis‐supranormal ejection fraction.

Subsequently, we conducted log‐rank tests using Kaplan–Meier curves for the four groups. The results remained consistent across the four groups, showing no significant differences in the primary composite outcome (log‐rank test; *p* = .14; Figure 2A) of all‐cause mortality (*p* = .90; Figure 2B). However, there was a statistical difference in HF hospitalization (*p* < .001; Figure 2C) among the groups.

Furthermore, while comparing patient characteristics, there was a difference in the proportion of men among the groups. To account for this influence, the analysis was restricted to women, and the results of the log‐rank test showed a similar trend (log‐rank test for HF hospitalization, *p* = .019; Supporting Information S1: Figure S1).

### 3.4 Cox hazard analysis among the four groups

**Figure 3:**
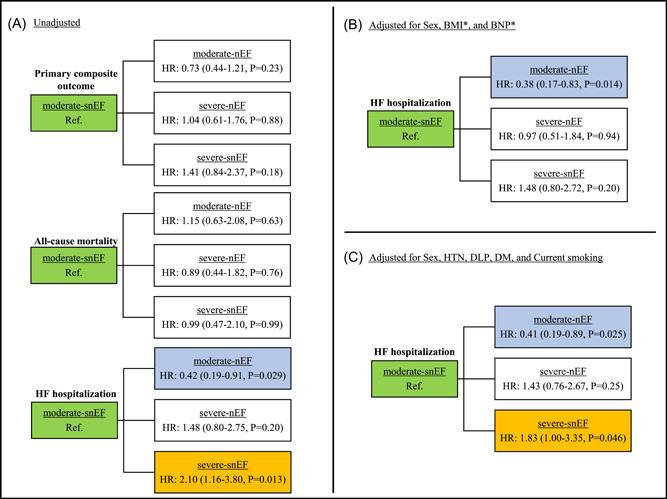
Univariate and multivariate cox hazard analysis for primary outcomes. (A) Unadjusted hazard ratio (HR) and 95% confidence interval. (B) HR adjusted for sex, log‐transformed brain natriuretic peptide level, and log‐transformed body mass index. (C) HR adjusted for hypertension, dyslipidemia, diabetes mellitus, and current smoking status. * Denotes a logarithmic transformation. Green, reference; white, no significant difference; blue or orange, statistically significant (*p* < .05).

In the Cox hazard analysis regarding the primary composite outcome, no significant differences were observed among the four groups (unadjusted hazard ratio [HR] with m‐snEF group as reference: m‐nEF group = 0.73, 95% confidence interval [CI] = 0.44–1.21; s‐nEF group = 1.04, 95% CI = 0.61–1.76; s‐snEF group = 1.41, 95% CI = 0.84–2.37; Figure 3A).

However, in the analysis of HF hospitalization, with the m‐snEF group as the reference, the adjusted HRs (adjusted for sex, BMI, and BNP levels) were as follows: m‐nEF group = 0.38, 95% CI = 0.17–0.83; s‐nEF group = 0.97, 95% CI = 0.51–1.84; and s‐snEF group = 1.48,95% CI = 0.80–2.72; Figure 3B).

Additionally, after adjusting for sex and atherosclerotic risk factors (hypertension, dyslipidemia, diabetes mellitus, and current smoking), the adjusted hazard ratios were as follows: m‐nEF group = 0.41, 95% CI = 0.19–0.89; s‐nEF group=1.43, 95% CI = 0.76–2.67; and s‐snEF group=1.83, 95% CI = 1.00–3.35 (Figure 3C).

### 3.5 Restricted cubic spline analysis

**Figure 4:**
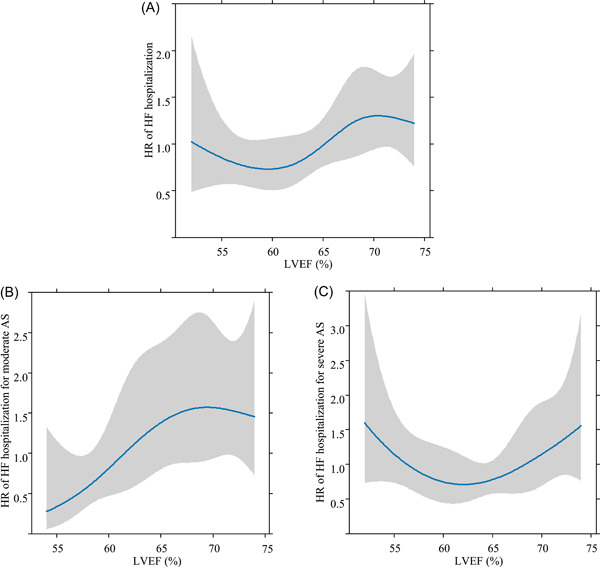
Relationship between heart failure hospitalization and left ventricular ejection fraction using restricted cubic spline analysis. (A) Relationship between the risk of HF hospitalization and LVEF using restricted cubic spline. (B) Moderate AS. (C) Severe AS. AS, aortic stenosis; HF, heart failure; HR, hazard ratio; LVEF, left ventricular ejection fraction.

Finally, using a restricted cubic spline analysis, we evaluated the relationship between the risk of HF hospitalization and LVEF (Figure 4A). As a result, in the analysis focusing on moderate AS, the risk of HF hospitalization peaked at LVEF between 65% and 70% (Figure 4B). Conversely, in cases of severe AS alone, a U‐shape relationship was observed, with a lower risk of HF hospitalization at an LVEF between 60% and 65% (Figure 4C).

## 4 DISCUSSION

This is the first study to compare the prognosis of snLVEF and nLVEF in patients with moderate to severe AS. The results indicated no significant difference regarding mortality, but an increased risk for HF hospitalization in patients with snLVEF. In the analysis considering severity, the risk of m‐nEF was lower than that of m‐snEF, the risk of s‐snEF was higher, but the comparison between m‐snEF and s‐nEF did not show a significant difference.

In recent years, there have been many reports of a U‐shaped relationship between mortality and high LVEF.^11^, ^13^, ^14^ Van Essen et al.^11^ evaluated mortality and hospitalization risks by stratifying HF patients based on EF. The results showed a tendency towards increased risk in a U‐shaped pattern, using LVEF 40%–49% as the reference point for 180‐day death. In our study, we did not observe a difference in the risk for all‐cause mortality between groups. This difference may be related to the characteristics of elderly AS patients, where 39%–45% of deaths are reported to be due to noncardiac diseases,^18^, ^19^ suggesting that noncardiac deaths may influence all‐cause mortality in elderly AS patients. In a report by Imamura et al., which included only patients with severe AS who underwent AVR, the risk of mortality was significantly higher in the snEF group than in the nEF group. Therefore, in populations where the risk of cardiovascular‐related mortality is high, such as those with severe AS, LVEF > 65% may suggest a potential risk of mortality, similar to other cardiovascular diseases.

On the other hand, the previously reported U‐shaped relationship between the risk of HF and high LVEF was consistent with this study in severe AS patients.^13^, ^20^

Based on these results, we speculated based on myocardial characteristics and cardiac mechanics, considering the relationship with afterload due to AS. Rosch et al.^21^ and Popovic et al.^22^ found differences in myocardial characteristics between patients with LVEF of 50%–60% and those with LVEF > 60%, with the group with LVEF > 60% showing less concentric remodeling and fibrosis. In addition, in patients with LVEF > 65%, left ventricular volumes (LVDd and LVDs) were smaller, and left ventricular diastolic stiffness increased, resulting in a leftward shift in the end‐diastolic pressure‐volume relationship.^22^ This difference may contribute to a reduction in preload reserve and an exaggerated response to elevated blood pressure, resulting in a significant increase in left ventricular end‐diastolic pressure (LVEDP).^21^, ^22^, ^23^ When snLVEF coexists with AS, it induces concentric remodeling due to afterload, causing not only left ventricular wall thickening but also further narrowing of the left ventricular cavity.^24^ Although SV analysis was not conducted in this study, a comparison of patient characteristics revealed that LVDd and LVDs were significantly decreased in the snEF group. Moreover, significant thickening of the left ventricular posterior wall was observed in the s‐snEF group, supporting the aforementioned speculation. As expected, easily changing LVEDP leads to an increase in left atrial pressure, causing pulmonary congestion and exacerbating HF,^25^ as evidenced by the higher frequency of HF hospitalization events observed relatively early in this study through echocardiography.

We discovered that moderate AS with snEF may have a prognostic value similar to that of severe AS by including patients with moderate or severe AS in our analysis. Khan et al. showed that even moderate AS is a poor prognostic factor for heart failure patients with LVEF < 50%,^9^ depending on the circumstances, even moderate AS can have a significant impact on patient prognosis. We believe that this study provides important findings to better define the target population for AVR in patients with moderate AS, which is recommended as Class IIA in the latest guidelines.^8^

### 4.1 Study limitations

This study had some limitations. First, this was a retrospective single‐center study, and the involvement of potential bias cannot be ruled out. Additionally, the small sample size and shorter follow‐up period compared to previous studies may have led to an underestimation of mortality events and inadequate statistical power. Furthermore, there were significant differences in the men‐to‐women ratio among the four groups in this study. Although analyses were conducted with maximum consideration of the influence of sex differences on the results, it is possible that this bias was not completely eliminated. Moreover, reliable data on patients' frequent or emergency visits due to worsening HF were not consistently obtained in this retrospective study. This lack of information could significantly impact the study and may lead to overinterpretation of the results in some cases. However, considering the concerns regarding information collection and reporting bias, this impact was not assessed.

## 5 CONCLUSION

In conclusion, compared to the m‐snLVEF group, there were fewer HF hospitalization in the m‐nLVEF group, while no significant difference was observed in comparison to the s‐nLVEF group. Thus, more intensive treatment may be beneficial in patients with moderate AS and snLVEF. However, to address these biases and generalize the results of this study, prospective and large‐scale studies are required.

## CONFLICT OF INTEREST STATEMENT

The authors declare no conflict of interest.

## Supporting information

Kaplan–Meier curves for the primary outcome in only women. (A) Kaplan–Meier curves for primary composite outcomes in four groups. (B) All‐cause mortality. (C) HF hospitalization. LVEF, left ventricular ejection fraction; m‐nEF, moderate aortic stenosis‐normal ejection faction; s‐nEF, severe aortic stenosis‐normal ejection fraction; m‐snEF, moderate aortic stenosis‐supranormal ejection fraction; s‐snEF, severe aortic stenosis‐supranormal ejection fraction; HF, heart failure.

Supporting information.

## Data Availability

The data that support the findings of this study are available on request from the corresponding author. The data are not publicly available due to privacy or ethical restrictions. The data in this article will be shared on reasonable request to the corresponding author.
