# Supplementary material for: Risk of supranormal left ventricular ejection fraction in patients with aortic stenosis
Source: Clin Cardiol. 2024 Mar 12;47(3):e24255. doi: 10.1002/clc.24255 (PMC10928761; doi:10.1002/clc.24255)
Supplement: Supplementary file 1 — Kaplan–Meier curves for the primary outcome in only women. (A) Kaplan–Meier curves for primary composite outcomes in four groups. (B) All‐cause mortality. (C) HF hospitalization. LVEF, left ventricular ejection fraction; m‐nEF, moderate aortic stenosis‐normal ejection faction; s‐nEF, severe aortic stenosis‐normal ejection fraction; m‐snEF, moderate aortic stenosis‐supranormal ejection fraction; s‐snEF, severe aortic stenosis‐supranormal ejection fraction; HF, heart failure. [file CLC-47-e24255-s002.pptx]

## Slide 1
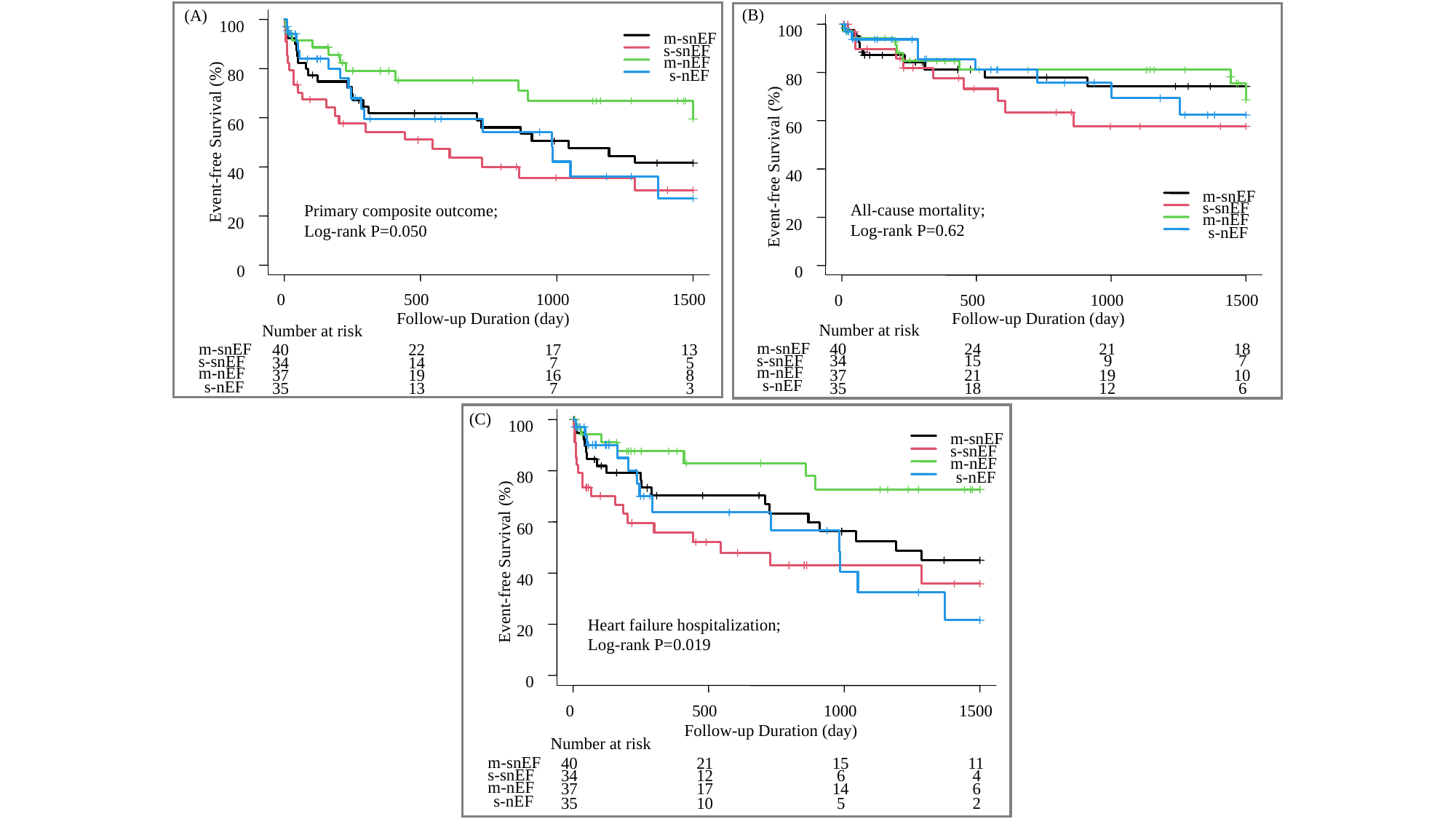

(B)
(A)
100
100
m-snEF
s-snEF
m-nEF
s-nEF
80
80
60
60
Event-free Survival (%)
Event-free Survival (%)
40
40
m-snEF
All-cause mortality;
Log-rank P=0.62
Primary composite outcome;
Log-rank P=0.050
s-snEF
m-nEF
20
20
s-nEF
0
0
0
500
1000
1500
0
500
1000
1500
Follow-up Duration (day)
Follow-up Duration (day)
Number at risk
Number at risk
m-snEF
40
24
21
18
m-snEF
40
22
17
13
s-snEF
34
15
9
7
s-snEF
34
14
7
5
m-nEF
m-nEF
37
21
19
10
37
19
16
8
s-nEF
s-nEF
35
18
12
6
35
13
7
3
(C)
100
m-snEF
s-snEF
m-nEF
s-nEF
80
60
Event-free Survival (%)
40
Heart failure hospitalization;
Log-rank P=0.019
20
0
0
500
1000
1500
Follow-up Duration (day)
Number at risk
m-snEF
40
21
15
11
s-snEF
34
12
6
4
m-nEF
37
17
14
6
s-nEF
35
10
5
2
