# Supplementary material for: Risk of supranormal left ventricular ejection fraction in patients with aortic stenosis
Source: Clin Cardiol. 2024 Mar 12;47(3):e24255. doi: 10.1002/clc.24255 (PMC10928761; doi:10.1002/clc.24255)
Supplement: Supplementary file 2 — Supporting information. [file CLC-47-e24255-s001.docx]

**Table S1.** The number of events and incidence rates for each group

| **Outcome** | **m-snEF group**  **(n = 67)** | | **s-snEF group**  **(n = 41)** | | **m-nEF group**  **(n = 65)** | | **s-nEF group**  **(n = 53)** | |
| --- | --- | --- | --- | --- | --- | --- | --- | --- |
|  | Events | Incidence rate (/100 person-year) | Events | Incidence rate (/100 person-year) | Events | Incidence rate (/100 person-year) | Events | Incidence rate (/100 person-year) |
| **Primary composite outcome** | 37 | 24.6 | 24 | 36.8 | 25 | 18.4 | 22 | 28.1 |
| **All-cause mortality**  **HF hospitalization** | 22  24 | 12.5  16.6 | 10  21 | 13.0  38.1 | 22  9 | 14.8  7.2 | 12  18 | 11.7  28.8 |

Data are presented as number of patients (n).

Abbreviations: HF, heart failure; m-nEF, moderate aortic stenosis-normal ejection fraction; m-snEF, moderate aortic stenosis-supra-normal ejection fraction; s-nEF, severe aortic stenosis-normal ejection fraction; s-snEF; severe-supra-normal ejection fraction.
